# Evaluation of Coordination of Emergency Response Team through the Social Network Analysis. Case Study: Oil and Gas Refinery

**DOI:** 10.1016/j.shaw.2014.09.004

**Published:** 2014-10-14

**Authors:** Iraj Mohammadfam, Susan Bastani, Mahbobeh Esaghi, Rostam Golmohamadi, Ali Saee

**Affiliations:** 1 Department of Occupational Hygiene, School of Public Health and Research Center for Health sciences, Hamadan University of Medical Sciences, Hamadan, Iran; 2 Department of Social Science, Al-Zahra University, Tehran, Iran; 3 Department of Social Science, Tarbiat – e- Modares University, Tehran, Iran

**Keywords:** cohesion, coordination, emergency response team, social network analysis

## Abstract

**Background:**

The purpose of this study was to examine the cohesions status of the coordination within response teams in the emergency response team (ERT) in a refinery.

**Methods:**

For this study, cohesion indicators of social network analysis (SNA; density, degree centrality, reciprocity, and transitivity) were utilized to examine the coordination of the response teams as a whole network. The ERT of this research, which was a case study, included seven teams consisting of 152 members. The required data were collected through structured interviews and were analyzed using the UCINET 6.0 Social Network Analysis Program.

**Results:**

The results reported a relatively low number of triple connections, poor coordination with key members, and a high level of mutual relations in the network with low density, all implying that there were low cohesions of coordination in the ERT.

**Conclusion:**

The results showed that SNA provided a quantitative and logical approach for the examination of the coordination status among response teams and it also provided a main opportunity for managers and planners to have a clear understanding of the presented status. The research concluded that fundamental efforts were needed to improve the presented situations.

## 1 Introduction

The emergency response team (ERT) is considered the most effective approach for dealing with emergencies in industries, and for minimizing the risk of casualties and losses. Several groups and team members with different levels of experience and different roles and responsibilities work together in the ERT. It is expected from them to respond to the emergency as effectively and reasonably as possible. This response depends on effective emergency preparedness of the groups and team members. It was also found that effective preparedness requires close coordination among each of the response teams. Effective coordination itself implies the understanding of team members regarding each other's roles and responsibilities before an emergency occurs [1,2]. This understanding is achieved through coordination of services and activities among different responding teams [3]. In fact, organizations require a comprehensive understanding of different sectors regarding their roles, responsibilities, and authorities [1,4]. This helps in scheduling tasks and ensuring the proper management of activities [5]. This kind of understanding is also essential to minimize duplication of services, to facilitate communication [6], and to allow responders to know about each other's activities in specific conditions when necessary. Ideally, it will also be useful in the division of responders' responsibilities, information sharing, mutual agreements, common planning and programs, and in the elimination of the gaps in services [7], which was recognized as a basic problem in Hurricane Katrina [8].

Lack of coordination is a challenge for the organizational performance of various groups [9,10], and has been recognized as a crucial hidden problem which has been principally ignored, and is accepted as one of the most important challenges that may lead to breakdowns to the response between teams [11], such as in large California wildfires [12]. Consequently, evaluations of coordination among response teams have gained increasing attention. Thus, before the response team enters an emergency and its members try to collaborate with each other, it is important to have an understanding of the coordination climate among teams and organizations, and to provide support, if necessary, in order to have an effective response. The purpose of this research as a case study is to conduct a quantitative assessment of the coordination status in ERT in a refinery. Therefore, the principle of *social network analysis* (SNA) was applied.

SNA is used to help decision makers and planners to identify the coordination status in different groups and organizations. Some researchers applied SNA to evaluate interorganizational relationships in medical sections. Studies have revealed that coordination of activities and development of relations are important issues in promoting medical and social services [13]. SNA has been used to assess interorganizational collaborations in the study of mental health systems [14]. In addition, in the emergency response following Hurricane Katrina, SNA techniques were successfully employed to find out the key members of multiorganizational coordination networks [15]. In response to catastrophic disasters, SNA assesses the relationship between responding organizations and their emergency coordination operations [16]. In the interorganizational network of responding to terrorist attacks, SNA identifies the major organizations that coordinate in the response system [17].

SNA studies and analyzes the relationships between different members and groups and structural characteristics within networks and it also provides a mathematical approach for measuring the strength of connections [18,19]. SNA plays a critical role in the determination of the degree of a team's success in achieving their goals and in the evaluation of the performance of the entire network [20]. It also plays an important role in the determination of the connections within and between parts of a network, which is known as cohesion [21–23], and it is considered an important characteristic of networks. This characteristic is measured by SNA indicators, such as density, degree, transitivity, and reciprocity, and an increase in each of these indicators enhances the cohesive level of network and improves coordination [22–25]. These indicators are explained below.

*Density* is a measure of the total connectedness of a network; it is the number of ties in a network as a ratio of all possible ties in a network and describes the general level of cohesion. The high value of density implies the strengthening of coordination between groups, and it increases the chance for social control of the network. If networks are denser or more cohesive, the score is 1 or 100%, which implies that all members in the network are directly connected together and a score of 0 shows that the network is entirely disconnected [21,23,25–27].

The *degree* is a measure that varies between the values of 0 and 1, with higher values representing a greater degree of centralization around one or a few members. In direct relations, the degree is divided into in- and out-degree centrality, implying the number of connections coming in and out to a given member, respectively. The high values of in- and out-degree in the whole network indicated that those limited numbers of central members have great reputations and influence other members [18,21,23,25,28]. It describes the extent to which this cohesion is organized around particular key members [23,26].

The level of *reciprocity* describes the degree to which a member has mutual connections to another member and it is an important indicator for stability of the network and development of trust among members. In direct relations with two members, if A coordinated with B, it is expected of B to have an increased tendency to coordinate with A, implying symmetric ties. This is a measure for the symmetric ties of a network. If this measure is near 0, it will correspond to low reciprocity, and values near 100% indicate high reciprocity [21,22,25].

*Transitivity* describes the tendency between two members in the network to be connected if they share a common mutual neighbor. In direct relations with three members, if A trusts B, and B trusts C, then A is likely to trust C without direct connections. If this measure is near 0, it will correspond to low transitivity, and values near 100% imply high transitivity, which is a key attribute of SNA [21,22,25].

## 2 Materials and methods

### 2.1 ERT

The present study was carried out in an ERT of a refinery that included seven teams composed of 152 team members, as listed in Table 1. All participants were male. Each team member undertook a set of roles and responsibilities formally assigned to him in the structure of the emergency management of the refinery. Important questions raised here are whether the response teams of the ERT have effective coordination together and if they are highly cohesive for ensuring effective response. In order to respond to these questions, the principle of SNA was utilized.

### 2.2 Data collection and analysis

The required data were collected through structured interviews (face to face) using a formal identification list including name, responsibility, and affiliation of each team member. Each member of the ERT was asked to choose those who they coordinated with. All selections were then recorded, archived, and analyzed as a whole network. Cohesion indicators of SNA such as density, degree, reciprocity, and transitivity were used to describe the structure of the network. This study uses binary data (absent, i.e., 0.0, and present, i.e., 1.0) and directional relations. The value is present if there be a selection within the team members. If each pair of team members does not select each other, the value of 0.0 will be allocated. The tie is directed from one member to another in a pair, i.e., it has an origin and a destination [21]. The connections for directed data are asymmetrical, because a directed line from members of rescue to firefighting will not necessarily involve a reciprocated line directed from members of firefighting to rescue. Therefore, the density was measured through grouping for each pairs of teams in two directions. The analysis and visualization of the survey are performed using UCINET (Version 6.0) SNA program (Analytic Technologies, Lexington, KY, USA) [29].

## 3 Results

### 3.1 Density

Density index was calculated as a whole network, between pairs of teams and each team separately. It is presented in Table 2. The results indicate that the density of each team individually is nearly desirable. This is presented in the main diameter of Table 2. Also, each of the cells in the table contains a measure of the density of the connections between pairs of teams. The rows and columns of the table display the value of the cohesion of a team with other teams and the cohesion of other teams with a specific team, respectively. For instance, the density between firefighting and rescue is 0.95, meaning that 95% of all possible relationships between members of firefighting and rescue members were established, and conversely, the possible relationship between members of rescue with firefighting was obtained to be 94%. This finding indicates that both teams have more dense networks than other teams. Collectively, the density of the whole network was 0.23, meaning that only 23% of all possible connections among members of the response teams are present (Table 2). This indicates that the actual ties among response teams are very limited, compared to the probable ties that may occur if coordination among teams is conducted optimally. This showed that the density level of the whole network was small.

### 3.2 Degree centrality

The findings showed that the ERT network had a relatively low in- and out-degree centralization index (Table 3) and that there were limited central members with enough reputation and influence in the network.

### 3.3 Reciprocity

The findings revealed that 87% of members in the ERT had reciprocated connections which imply a high value of mutual relations (Table 3), i.e., most team members had a tendency to make mutual coordination with each other and were equally interested in keeping up their coordination.

### 3.4 Transitivity

The results also indicated that 35.42% of members in the ERT had triple connections that imply low values of triplet relations (Table 3). This means that if Fire-O-K and Fire-O-B have coordinated together and also Fire-O-K and HSE-S-M have coordination and tie together, there will be a low tendency to have coordination among Fire-O-B and HSE (Health, Safety and Environment)-S-M.

## 4 Discussion

The goal of the ERT is to reach high performance which is obtained through coordination of activities and services among response teams, prior to involvement in the actual operations. In emergency management we generally face several teams that can be defined as a network, the members of which cooperate based on relations and interactions. This issue can be examined through measuring social network indicators. In this study, cohesion of coordination ties was measured by SNA indicators including density, degree, reciprocity, and transitivity in the whole network of the ERT. Each index has a different interpretation. The interval of defined indicators is between 0 and 1 based on what we can decide about the overall status of the network according to quantitative results of indices (in 0 and 1 intervals) and considering experts' opinions. High coordination can be achieved when the density is high, and the percentage of binary and triple bonds for the increase of performance is high, and vice versa. By increasing each of these indicators, the cohesion of the network will increase and it will consequently facilitate the coordination among response teams, which plays an important role in the emergency management.

Structural characteristics of a network, such as density, are obtained through connections and cohesions between teams, which can be used to interpret their performance [25]. To determine the level of cohesion in the ERT network, density was calculated in three levels including inter-team, between pairs of teams, and the whole ERT network separately. At the inside level of each team, the results showed an acceptable value of cohesion, except for the HSE and logistic teams. This was due to the variety of units and members of these teams which also affected the results. In addition, the findings show that there are various cohesions from low to high values among the response teams. According to the results, approximately 33.33% of connections are entirely disconnected. A high percentage of this value is allocated to the public relations which operates as an independent unit and does not have an active role in the coordination network. Then, the logistic and security teams showed low cohesions. The findings revealed that three response teams (firefighting, rescue, and HSE) had moderate cohesions with the medical team but the medical team itself had a low dense network in relation to other teams, especially with the rescue team. Due to various units with different responsibilities, the HSE team acts as a mediator that can be used to improve the coordination situations of the ERT. Also, firefighting and rescue teams, due to having a high level of cohesion, can play a key role in creating sustainable ties among teams, which could enhance coordination during normal operations as well as during emergencies that require essential actions. Finally, the results of the density showed that the cohesion of the whole network is very low. This means that the team members of the response team are weakly connected to coordinate together, which is a challenge for emergency management and needs efforts to reach a dense coordination among response teams.

High in-degree of centralization showed that a limited number of central members in the network received more confidence from other members and had a great reputation in terms of coordination in the network. Also high out-degree indicated that the central members in terms of coordination had a great influence on the network. The results of the centralization index showed a low level for both degrees and indicated that the percentage of the ties that were controlled by or depended on key members in the network was relatively low. This finding was supported by previous researches [24,25].

Reciprocity and transitivity are essential indicators for stability of a network which is used to determine and judge about the stability and cohesion of most social networks [24,25]. The findings of the study showed that team members had a high level of mutual relationships, which promotes more stable and cohesion ties within the response team and is beneficial for coordination. When one member builds coordination with two other members in a response team, it means that they tend to coordinate together and create denser ties in the ERT. Some research conducted in the field of management has confirmed this finding [25,30]. Also, transitivity examined three members and the level of coordination relations between them. The findings revealed a reduction in triple relations compared to the mutual connections. This implies low stability of the connections and that the ERT had difficulty in coordinating activities and resources. The results of local collaboration networks and sustainable development study support this finding [24]. Generally, the results showed a high level of mutual ties with low transitivity in the coordination network. Previous studies have shown that networks with high reciprocity and high in-degree for relationships between the team members are indicators of high team performance [25,31]. In addition, it was found that networks with high transitivity were more cliquish than those with low transitivity and members tend to standardize performance and actions in the structure of their own team (in a network with high density) [22,25]. These findings are inconsistent with the results of this study (low density, high reciprocity, low in-degree, and low transitivity).

In fact, with regard to the low number of cliques, poor relationships with key members, and high levels of mutual relations in the network with a low density of ties, it can be concluded that there are low cohesions of coordination network in the ERT. These results allow planners and managers to have a clear understanding of the presented status and to make better efforts to enhance and strengthen the situation. The creative approaches, such as the participatory training, drills, and maneuvers, along with need assessment and standards are emphasized in order to acquire an appropriate level of coordination and to ensure that all team members of a response team have an opportunity to become familiar with each other's responsibilities and activities. This could be beneficial in improving the level of coordination and the emergency response consequently. It is recommended that these programs be evaluated on a regular basis.

In summary, the present research demonstrates that SNA provides a logical and qualitative approach to examine the status of coordination among response teams in the ERT. The most obvious finding that emerged from this study was that the response teams have a relatively low percentage of coordination. The research concludes that emergency management has been revised toward the existing training programs and has used creative programs to improve the presented situations which require fundamental efforts along with evaluation of the effectiveness of programs.

## Conflicts of interest

No potential conflicts of interest relevant to this article were reported.

## Figures and Tables

**Table 1:** The structure of the emergency response team

No.	Response team	Composition
1	Firefighting	Supervisors (1), assistance (1), officers (4), firefighters (20), mechanics (2), drivers (7)
2	Rescue	Supervisors (1), rescue (3), drivers (1)
3	HSE	Managers (1), assistance (1), clerks (1), safety (10), permit (2), HSEMS (2), health (5), traffic (9), electrical (1), machine (2)
4	Medical	Supervisors (1), doctors (4), nurses (6), pharmacists (2), services (2), reception (2), drivers (3)
5	Logistic	Supervisors (1), assistance (1), maintenance and repairs (11), storekeepers (5)
6	Security	Supervisors (1), assistance (1), control (13), physics or operational (19)
7	Public relations	Supervisors (1), assistance (1), employees (4)

HSE (Health, Safety and Environment); HSEMS (Health, Safety and Environmental Management System).

**Table 2:** The results of density index of response teams in the emergency response team (ERT)

Total = 0.23	Firefighting	Rescue	HSE	Medical	Logistic	Security	Public relations
Firefighting	0.98	0.95	0.23	0.19	0.07	0.02	0
Rescue	0.94	1	0.234	0.44	0	0	0
HSE	0.23	0.27	0.580	0.25	0.086	0.124	0
Medical	0.1	0.007	0.224	0.92	0	0	0
Logistic	0.07	0	0.084	0.05	0.4	0.04	0
Security	0.02	0	0.43	0.016	0.04	0.705	0.093
Public relations	0	0	0	0	0	0.093	1

HSE (Health, Safety and Environment).

**Table 3:** The results of social network analysis (SNA) indicators of the coordination network

Indicator	(%)
Degree centrality	
In	31.58
Out	28.23
Reciprocity	87
Transitivity	35.42
